# Ezrin/Radixin/Moesin Proteins and Flotillins Cooperate to Promote Uropod Formation in T Cells

**DOI:** 10.3389/fimmu.2013.00084

**Published:** 2013-04-08

**Authors:** Sibylla Martinelli, Emily J. H. Chen, Fiona Clarke, Ruth Lyck, Sarah Affentranger, Janis K. Burkhardt, Verena Niggli

**Affiliations:** ^1^Department of Pathology, University of BernBern, Switzerland; ^2^Department of Pathology and Laboratory Medicine, Children’s Hospital of Philadelphia and The Perelman School of Medicine, University of PennsylvaniaPhiladelphia, PA, USA; ^3^Theodor Kocher Institute, University of BernBern, Switzerland

**Keywords:** ezrin, radixin, moesin, T-lymphocyte, flotillin, cell polarization, cell migration

## Abstract

T cell uropods are enriched in specific proteins including adhesion receptors such as P-selectin glycoprotein ligand-1 (PSGL-1), lipid raft-associated proteins such as flotillins and ezrin/radixin/moesin (ERM) proteins which associate with cholesterol-rich raft domains and anchor adhesion receptors to the actin cytoskeleton. Using dominant mutants and siRNA technology we have tested the interactions among these proteins and their role in shaping the T cell uropod. Expression of wild type (WT) ezrin-EGFP failed to affect the morphology of human T cells or chemokine-induced uropod recruitment of PSGL-1 and flotillin-1 and -2. In contrast, expression of constitutively active T567D ezrin-EGFP induced a motile, polarized phenotype in some of the transfected T cells, even in the absence of chemokine. These cells featured F-actin-rich ruffles in the front and uropod enrichment of PSGL-1 and flotillins. T567D ezrin-EGFP was itself strongly enriched in the rear of the polarized T cells. Uropod formation induced by T567D ezrin-EGFP was actin-dependent as it was attenuated by inhibition of Rho-kinase or myosin II, and abolished by disruption of actin filaments. While expression of constitutively active ezrin enhanced cell polarity, expression of a dominant-negative deletion mutant of ezrin, 1–310 ezrin-EGFP, markedly reduced uropod formation induced by the chemokine SDF-1, T cell front-tail polarity, and capping of PSGL-1 and flotillins. Transfection of T cells with WT or T567D ezrin did not affect chemokine-mediated chemotaxis whereas 1–310 ezrin significantly impaired spontaneous 2D migration and chemotaxis. siRNA-mediated downregulation of flotillins in murine T cells attenuated moesin capping and uropod formation, indicating that ERM proteins and flotillins cooperate in uropod formation. In summary, our results indicate that activated ERM proteins function together with flotillins to promote efficient chemotaxis of T cells by structuring the uropod of migrating T cells.

## Introduction

T-lymphocytes are highly motile cells that are crucial for rapid recognition of foreign antigens and destruction of virally infected cells or tumor cells. T cell motility is indispensable for fulfilment of these functions. T cell polarization and directional migration are complex processes that are not well understood. Upon stimulation with chemokines, T-lymphocytes rapidly develop a contracted tail (uropod) and an F-actin-rich front with protrusions. T cell front-tail polarity is a prerequisite for efficient crawling. Polarization and migration require a functional cytoskeleton, reversible actin polymerization in the front, and myosin-dependent contractility in the rear (Krummel and Macara, 2006; Ley et al., 2007; Friedl and Weigelin, 2008). We are interested in unraveling mechanisms involved in development of self-organizing front-tail polarity in leukocytes.

Development of front-tail polarity in leukocytes requires segregation and activation of specific signaling and cytoskeletal molecules in the retracting rear and protrusive leading edge of the cells. This process is independent of an external gradient of chemoattractant. Localized positive feedback loops and inhibitory effects of front signaling pathways on rear signaling and vice versa are thought to reinforce this biochemical and structural cell polarization (Bagorda and Parent, 2008). Specific proteins, including adhesion receptors, cytoskeletal proteins, and membrane-microdomain scaffolding proteins such as flotillin-1 and -2, have been shown to segregate to the uropod of polarizing T-lymphocytes (Sánchez-Madrid and Serrador, 2009; Affentranger et al., 2011; Baumann et al., 2012). Localized Rho activation in the rear results in uropod contraction and suppresses formation of protrusions; localized Rac activation in the front induces formation of F-actin-rich protrusions. The T cell uropod has been implicated in migration through constricted spaces and may mediate recruitment of other cells (Serrador et al., 1997; Sánchez-Madrid and Serrador, 2009; Soriano et al., 2011). We previously have shown that flotillins are involved in T cell uropod formation (Affentranger et al., 2011). In this work, we focus on the role of the F-actin-plasma membrane linkers ezrin/radixin/moesin (ERM) in T cell uropod formation, and ask how this relates to that of flotillins.

The closely related ERM proteins are ubiquitously expressed in mammalian tissues. They occur in a dormant and an activated form. Interaction with phosphatidylinositol 4,5-bisphosphate (PIP-2) induces conformational changes in ERM proteins, which subsequently allow C-terminal phosphorylation for stable activation. PIP-2 is required in T cells for continuous membrane association and maintenance of the phosphorylated form of the ERM proteins (Barret et al., 2000; Fievet et al., 2004; Hao et al., 2009; Ben-Aissa et al., 2012). Activated ERM proteins bind to F-actin with their C-terminus and to cytoplasmic tails of transmembrane proteins or protein adaptors with their N-terminal FERM domain, thus connecting the cortical F-actin network to the plasma membrane (Niggli and Rossy, 2008; Neisch and Fehon, 2011). Human T-lymphocytes express mainly moesin and ezrin. In resting T cells, ERM proteins are strongly phosphorylated and associated with the plasma membrane. Chemokine activation of human T cells is accompanied by a transient partial dephosphorylation of ERM proteins and partial dissociation from the plasma membrane (Brown et al., 2003; Hao et al., 2009). Later, C-terminally phosphorylated ERM (P-ERM) proteins accumulate in the uropod of chemokine-stimulated T cells, recruiting transmembrane adhesion receptors such as PSGL-1 to this site and colocalizing with the lipid raft-associated flotillins (Lee et al., 2004; Sánchez-Madrid and Serrador, 2009; Affentranger et al., 2011).

Conflicting data have been published on the role of ERM proteins in chemokine-induced polarization and migration of T-lymphocytes. In studies with a murine T lymphoma cell line and with naïve human T cells, overexpression of the phosphomimetic, constitutively active mutant ezrin T567D [but not of wild type (WT) ezrin] has been reported to increase the size of the uropod and enhance chemokine-induced chemotaxis (Lee et al., 2004; Li et al., 2007). In human T-lymphoblasts, transfection with constitutively active moesin reverted inhibition of polarization and motility mediated by constitutively active Rac (Cernuda-Morollon et al., 2010). Conversely, transfection with the N-terminal part of ezrin (aa 1–366) partially reduced the fraction of T cells with uropods (Lee et al., 2004). The latter deletion mutant is thought to act as a dominant-negative mutant, by competing with endogenous ERM proteins for binding sites on receptors, thus uncoupling the receptors from the cortical actin network (Martin et al., 1995; Faure et al., 2004). Lee et al. (2004) propose that ERM proteins accumulate at the site of the future uropod and locally activate Rho, resulting in formation of a contracted uropod. In line with these findings, naïve T cells derived from mice lacking moesin show reduced *in vitro* chemotaxis to CXCL12 and CCL21 (Hirata et al., 2012). Moreover murine T-lymphoblasts lacking ezrin and with strongly reduced moesin expression chemotax less efficiently in response to CCL19 than WT cells through 3 μm pores in transwell assays (Chen et al., 2013).

In contrast to these data, Brown et al. (2003) observed that expression of constitutively active moesin T558D in *ex vivo* human T cells delayed SDF-1-induced cell polarization and inhibited resorption of microvilli. Liu et al. (2012) reported that T-lymphoblasts isolated from mice expressing phosphomimetic ezrin T567E specifically in T cells show attenuated *in vitro* migration and chemotaxis and *in vivo* homing and transmigration, as well as reduced lamellipod extension, as compared to cells overexpressing WT ezrin. The attenuation of protrusion in these cells was attributed to increased membrane tension due to increased actin-membrane linkage via T567E ezrin.

We have now attempted to clarify the role of ERM proteins in T cell polarization, uropod scaffolding, and migration using expression of WT, constitutively active and dominant-negative ezrin proteins. Our data clearly support a positive role for ERM proteins in T cell polarization and migration. Our results also suggest that ERM proteins and flotillins mutually promote their uropod capping and thus cooperate in uropod formation.

## Materials and Methods

### Materials and suppliers

Stromal cell-derived factor 1 (SDF-1 = CXCL12): Peprotech. Latrunculin A: Alexis Biochemicals. Blebbistatin: Enzo Life Sciences. Y-27632: Calbiochem. Bovine serum albumin (BSA): Serva. Lysolecithin (l-α-lysophosphatidylcholine): Sigma. Hoechst 33342: Sigma-Aldrich. Gey’s solution contained 138 mM NaCl, 6 mM KCl, 100 μM EGTA, 1 mM Na_2_HPO_4_, 5 mM NaHCO_3_, 5.5 mM glucose, and 20 mM HEPES (pH 7.4).

### Antibodies

A polyclonal anti-CD3 antibody (Cat. No. RM-9107) was obtained from NeoMarkers. Polyclonal antibodies directed against moesin (Cat. No. 3150), ERM (Cat. No. 3142), and phospho ezrin (Thr567)/radixin (Thr564)/moesin (Thr558) (Cat. No. 3141) were from Cell Signaling Technology. Polyclonal antibodies raised in rabbits against full-length human recombinant ezrin and against the recombinant N-terminal domain of ezrin (Andreoli et al., 1994) were kindly provided by P. Mangeat (Université Montpellier II, France). A polyclonal antibody specifically recognizing β-cytoplasmic actin was kindly provided by C. Chaponnier (Dugina et al., 2009). Monoclonal murine antibodies directed against flotillin-2 (Cat. No. E35820) and PSGL-1 (Cat. No. 556053) were obtained from Transduction Laboratories/BD Pharmingen, Germany. The Alexa 488-conjugated goat-anti-rabbit (Cat. No. A11008) and Alexa-568-conjugated goat anti-mouse IgG antibodies (Cat. No. A11001) were from Molecular Probes.

### Constructs

Constructs encoding WT full-length human ezrin tagged at its C-terminus with EGFP (WT ezrin) and a dominant-negative deletion mutant of human ezrin (aa 1–310) C-terminally tagged with EGFP were kindly provided by Lamb et al. (1997). Ezrin cloned into the plasmid pEGFP-N1 was used as a PCR template to generate the constitutively active mutant ezrin T567D. The single-point mutation was inserted by PCR and the products were cloned into the vector pEGFP-N1 (ClonTech Laboratories) (primer for the mutation: ggacaagtacaaggacctgcggcagatcc). Constructs encoding flotillin-1 and -2 C-terminally tagged with mCherry were generated as described previously (Rossy et al., 2009).

### Isolation of T-lymphocytes

Human T cells were isolated from buffy coats using the Pan T Cell Isolation Kit II (Miltenyi Biotec) and separation on LD columns (Miltenyi Biotec) according to the manufacturers instructions. Briefly, mononuclear cells obtained from buffy coats as described (Affentranger et al., 2011) were incubated with a cocktail of biotin-conjugated antibodies against CD14, CD16, CD19, CD36, CD56, CD123, and CD235a (glycophorin A). Cells binding these antibodies were subsequently depleted from the cell suspension using anti-biotin MicroBeads and magnetic separation. The resulting cell suspension contained >95% T-lymphocytes as assessed using anti-CD3 staining. The cells were used after overnight incubation in RPMI with 10% FCS at 37°C and 5% CO_2_.

To generate primary murine T cells, peripheral lymph nodes were harvested from C57Bl/6 mice (Jackson Labs). CD4+ T cells were isolated by negative selection using anti-CD8 (2.43) and anti-MHCII (M5/114.15.2). T cells were stimulated in 24-well plates coated with 1 μg/ml anti-CD28 (PV1) and anti-CD3 (2C11) (both from BioXCell) for 3 days and maintained in DMEM supplemented with 5% FBS, penicillin, streptomycin, GlutaMAX, Hepes, NEAA, and β-mercaptoethanol (all from Invitrogen) at 37°C, 10% CO_2_. T-lymphoblasts were then rested for 2 days before siRNA suppression.

### siRNA-mediated protein suppression and chemokine stimulation of murine T cells

SiRNA duplexes against mouse flotillin-2 were purchased from Dharmacon RNAi Technologies. SiGenome non-targeting control siRNA #2 was used as a control. Cells were washed and resuspended in unsupplemented DMEM. T cells (10^7^) were mixed with 400 pmol of either control or flotillin-2 siRNA in 4 mm gap cuvettes and transfected using a BTX ECM830 electroporator at 290 V for 10 ms. After transfection, T cells were maintained in supplemented DMEM and 100 U/ml rhIL-2 (obtained through the AIDS Research and Reference Reagent Program, Division of AIDS, NIAID, NIH from Dr. Maurice Gately, Hoffmann-La Roche Inc.) for 48–72 h. For chemokine stimulation, 48 h after electroporation, cells were resuspended in PBS and warmed to 37°C for 15 min. 10 nM SDF-1α (Peprotech), was added and cells were incubated at 37°C for 15 min prior to lysis at 4°C.

### Immunoblotting of T cells

Murine T cells were lysed at 10^8^ cells/ml in 1× RIPA buffer (150 mM NaCl, 50 mM Tris pH 7.5, 1% NP40, 0.5% Na-Deoxycholate, 10% glycerol, 0.1% SDS, 50 mM NaF, 1× Complete mini protease inhibitors, and 1 mM NaVO_4_) for 20 min on ice. Insoluble material was pelleted at 13,000 rpm for 20 min. Cells were boiled in sample buffer and separated on a 10% acrylamide Tris-glycine gel using SDS-PAGE electrophoresis. Proteins were transferred to nitrocellulose and blocked in 5% BSA in PBS. Blots were probed with antibodies specific for flotillin-1 or flotillin-2 (BD Biosciences), total ERM proteins (BD Biosciences anti-moesin), phospho-ERM proteins (Cell Signaling), and GAPDH (Millipore) in TBST, followed by AlexaFluor680 donkey anti-mouse IgG (Invitrogen) or IR800 goat-anti-rabbit IgG (Rockland). Proteins were visualized and quantified using the Odyssey Imager (LI-COR).

Human T cells were precipitated with trichloro acetic acid (TCA), separated on a 10% gel and subjected to immunoblotting. Blots were probed with antibodies specific to total ERM proteins or phospho-ERM proteins (Cell Signaling), followed by a goat-anti-rabbit IgG antibody coupled to horseradish peroxidase (BioRad) as described (Rossy et al., 2009). Proteins were visualized using an ECL Western detection system, detected with the LAS-4000 imaging system (Fujifilm) and quantified with the Quantity one system (BioRad).

### Transient expression of proteins in human T cells

For transfections, 3–5 × 10^6^ freshly isolated T-lymphocytes were resuspended in 100 μl human T cell nucleofector solution (Lonza) diluted 1:2 with PBS and 2–4 μg of plasmid DNA were added. The cell suspension and the plasmid DNA were then transferred to a cuvette and nucleofection was carried out (Amaxa Nucleofector, program U-14). Immediately, 500 μl of medium with 20% FCS was added and the T cells were transferred to a prewarmed 12-well plate containing 2.5 ml of medium with 20% FCS, followed by incubation at 37°C in a CO_2_ incubator for 4 h. Transfected T cells were subsequently washed, resuspended in Gey’s solution and used for experiments. In order to assess expression levels of ezrin constructs, the viable EGFP-positive cells were separated from the viable non-fluorescent and dead cells using an ARIA III cell sorter (BD Biosciences). The sorted cells were precipitated with TCA and subjected to immunoblotting as described (Rossy et al., 2009).

### Immunofluorescence staining

Human T cells: cells were incubated in suspension in plastic tubes in Gey’s buffer at 37°C with agitation (750 rpm) without or with stimuli (4–8 × 10^6^ cells/ml, 500 μl per assay) as indicated in the Figure legends. After incubation cells were fixed with 3.7% (final) paraformaldehyde (PFA) for 10 min at 37°C for visualization of tagged ezrin constructs and for β-actin, or with TCA for visualization of endogenous flotillin-2, ezrin, moesin, and P-ERM, as described (Affentranger et al., 2011). The fixed T cells were washed with PBS, cytocentrifuged, permeabilized with methanol (−20°C, 3–5 min) for detection of β-actin and washed with PBS. Cells were then incubated with the indicated primary antibodies followed by incubation with the appropriate secondary antibodies (Alexa 488-conjugated goat-anti-rabbit or goat anti-mouse IgG antibodies) as described (Affentranger et al., 2011). Images were taken with an Olympus Fluoview FV1000-IX81 confocal laser scanning microscope, using a 60× oil immersion objective. ERM-, flotillin-, and PSGL-1-enriched aggregates located at the plasma membrane of at least 1 μm or larger (maximally approximately 4 μm) were defined as “caps.” For analysis of shape and protein localization, 50–100 T cells were analyzed per sample in all experiments.

Murine T cells: 12 mm round glass coverslips were acid washed with 10% H_2_O_2_ in 0.1N HCL, rinsed in ddH_2_O followed by methanol, and flame dried. Coverslips were coated with 50 μg/ml of human fibronectin (R&D Systems) for 2 h at RT. T cells (1.5 × 10^5^) were resuspended in 50 μl unsupplemented DMEM, added to each coverslip and allowed to settle in a humidified chamber for 30 min at 37°C. Unbound cells were washed off and bound T cells were fixed using 3% PFA/PBS and quenched with 50 mM ammonium chloride. To quantify uropod formation, at least 50 randomly selected cells per coverslip were scored based on an elongated shape and the presence of a distinct tail-like structure. For staining of moesin, cells were permeabilized with 0.1% Triton X-100 after fixation, blocked with 2% horse serum in PBS and probed with rabbit anti-moesin, followed by Alexa Fluor 488 goat-anti-rabbit (Invitrogen). Cells were imaged using a Zeiss Axiovert 200M microscope with a 63× objective. ERM polarity was scored based on ERM localization in the uropod of polarized cells, or to an area less than one third of the cell circumference of unpolarized cells.

### 2D migration of human T cells on endothelial cells

Four to five days prior to the experiments, immortalized human cerebral microvascular endothelial cells (hCMEC/D3) (Weksler et al., 2005) at passage 32 were seeded on a 96 well plate (Greiner Bio-one, catalog no 655090) (4 × 10^4^ cells per well) in EBM-2 culture medium from Lonza (with 5% FCS, 1% Penicillin/Streptavidin, 1.4 μM Hydrocortisone, 5 μg/ml Acid Ascorbic, 0.01% chemically defined lipid concentrate, 10 mM Hepes, 1 ng/ml basic FGF). 20 h prior to the experiment hCMEC/D3 cells were stimulated with 20 ng/ml recombinant interleukin-1β (IL1β) (PromoKine, Vitaris). 30 min prior to the experiment hCMEC/D3 were washed with culture medium to remove IL1β.

For live-cell imaging 750,000 T cells, resuspended in 75 μl RPMI-Hepes with 10% FCS, were pipetted into a well of a 96 well plate containing 125 μl EBM-2 with supplements and a confluent monolayer of IL1β stimulated hCMEC/D3. T cells were allowed to settle for 10 min at 37°C. Then, the 96 well plate was mounted on the heated stage of an inverted microscope (AxioObserver.Z1, Carl Zeiss). Microscopic image acquisition was computer controlled through the AxioVision 4.2 software (Carl Zeiss), using the 20×/0.4 LD Plan-Neofluar objective. Images were taken with a high resolution monochrome AxioCam RMm Rev Camera (four images per min) for a time period of 10 min. For each condition two consecutive image series were acquired at different positions in the same well. Movies were created with eight frames per second (1 s of movie = 2 min real time). For evaluation of the % of migrating cells, the initial position of transfected T cells adhering to the endothelial cells was outlined, and only cells that had completely left the initial outline were scored as migrating. The speed of the migrating T cells was determined using Image J.

### Transwell chemotaxis

300,000 freshly isolated human T cells were transiently transfected with EGFP or the indicated ezrin constructs and resuspended in 0.1 ml RPMI (lacking FBS) containing 1% BSA. The cells were placed in Costar filter inserts with 3 μm pore size and allowed to migrate to the lower wells containing 0.6 ml RPMI and 1% BSA with or without SDF-1 (200 ng/ml) for 1 h at 37°C and 5% CO_2_. The % of migrated transfected T cells was determined by PFA fixation of an aliquot of the input sample and of the cells recovered in the lower wells and determination of the total cell numbers and the % of transfected cells in each sample.

### Fluorescence recovery after photobleaching experiments

Human T-lymphocytes were transfected with WT or mutant ezrin constructs and both flotillin-1 and -2 tagged with mCherry, followed by a 4 h incubation at 37°C in a CO_2_ incubator and a 15 min incubation in the absence or presence of 40 ng/ml SDF-1 in Gey’s medium containing 1 mM MgSO_4_, 1.1 mM CaCl_2_, and 5% BSA. A 5 μl aliquot of cell suspension (10 × 10^6^ cells/ml) was then immediately placed on a glass slide, covered with a glass coverslip and sealed with hot paraffin. Live-cell confocal imaging was performed at room temperature with a confocal laser scanning microscope Olympus Fluoview FV1000-IX81, 60× oil immersion objective. A picture of the cells was taken 2 s before bleaching of the EGFP moiety. A circular region of interest (ROI) of 2.5 μm in diameter in the region of the flotillin-enriched cap (if present) was continuously bleached at 8% of full laser power (473 nm for EGFP) for 12 s. Seven seconds after the completion of bleaching the first frame was taken. Fluorescence recovery was observed for 140 s (20 frames) at 0.5–1% laser power. For analysis the fluorescence of the bleached ROIs, corrected for the reduction in fluorescence of control ROIs located outside of the bleached area in the same cell which occurs due to image capture, and normalized to the initial fluorescence in the ROIs before bleaching, was plotted against time. The mobile fraction was calculated after correction for bleaching of the control ROIs according to *M*_F_ = (*F*_FR_ − *F*_0_)/*F*_i_ − *F*_0_) where *F*_FR_ is the average fluorescence after full recovery, *F*_0_ is the fluorescence immediately after the bleach, and *F*_i_ is the fluorescence just before photobleaching.

### Statistical analysis

Data were analyzed with the Graph Pad Prism software (version 5.04) using ANOVA with Tukey’s *post hoc* testing or, for the fluorescence recovery after photobleaching (FRAP) data, the Kruskal–Wallis test with Dunn’s multiple comparison test, or for the siRNA data, with the paired two-tailed *t*-test. *P* values < 0.05 were considered significant. Data correspond to the mean ± SEM.

## Results

### Localization of endogenous ERM proteins and flotillins in human freshly isolated T cells

**Figure 1:**
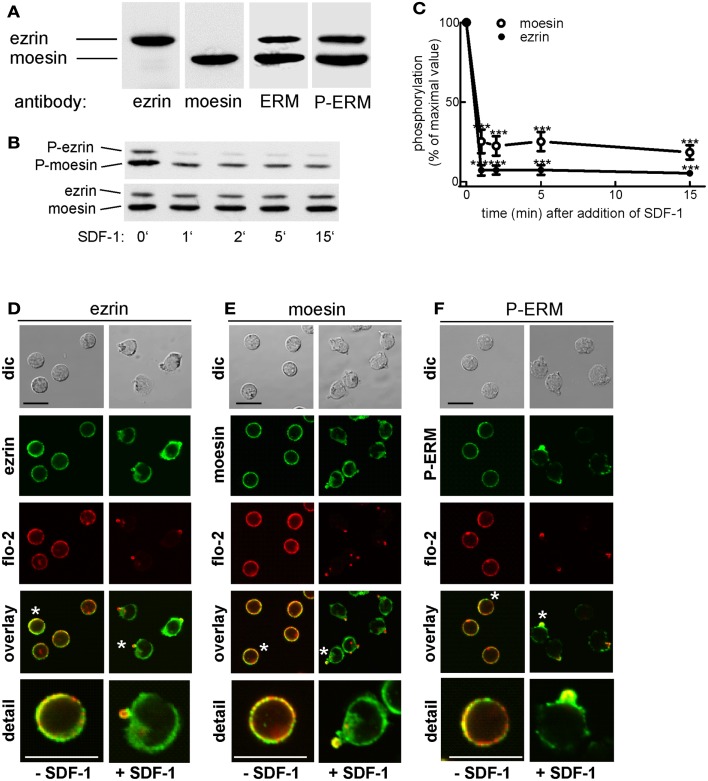
**Expression and localization of endogenous ERM proteins in human T cells**. **(A)** Freshly isolated human T cells (1 × 10^6^ cells per lane) were precipitated with TCA and subjected to immunoblotting (7.5% gel) using polyclonal rabbit antibodies directed against ezrin, moesin, all ERM proteins, or P-ERM, as indicated. **(B)** T cells were incubated in the absence or presence of 40 ng SDF-1/ml for the specified times, followed by precipitation with TCA and immunoblotting using an antibody recognizing P-ERM, and as loading control, an antibody recognizing all ERM proteins independent of phosphorylation. **(C)** Quantitative evaluation of the experiment shown in **(B)** obtained by densitometry (ratio of values obtained for phosphorylated moesin or ezrin, divided by values obtained for total moesin or ezrin). Mean ± SEM of four independent experiments. ***P < 0.001 for statistically significant differences to cells not exposed to SDF-1. **(D–F)** T cells were preincubated for 15 min at 37°C in the absence of stimuli, followed by a further incubation for 15 min in the absence or presence of 40 ng/ml SDF-1 as indicated. T cells were then fixed and stained for ezrin **(D)**, moesin **(E)**, or P-ERM **(F)** using the same antibodies as in **(A)**, and for flotillin-2 using monoclonal antibodies, followed by confocal microscopy (dic: differential interference contrast). Asterisks indicate cells that are shown enlarged in the panel at the bottom. Bar, 10 μm.

As a first step toward understanding how ERM proteins and flotillins function to promote uropod organization, we asked where endogenous ERM proteins are located in freshly isolated T cells as compared to flotillins and to what extent their location correlates with that of the activated, P-ERM proteins. Human freshly isolated T-lymphocytes express mainly ezrin and moesin (approximately 20 and 80% of total ERM proteins, respectively). Both ezrin and moesin react with an antibody selective for P-ERM in immunoblots (Figure 1A). In agreement with previous data obtained with freshly isolated human T cells (Brown et al., 2003), stimulation with SDF-1 results in rapid (but not complete) dephosphorylation of moesin and ezrin, down to 18 and 5% respectively of the values prior to addition of chemokine (Figures 1B,C). We analyzed the localization of endogenous total and P-ERM proteins and of flotillins in human T cells. Endogenous ezrin and moesin show mainly a random punctate cortical location in resting T cells, partially colocalizing with endogenous flotillin-2 (Figures 1D,E). In chemokine-stimulated cells, ezrin and moesin are present in the uropod, but also show a diffuse location, in contrast to endogenous flotillin-2, which is almost exclusively located in the uropods (Figures 1D,E). Staining of resting cells with an antibody reacting exclusively with phosphorylated, activated ERM proteins also indicates a random cortical location. Upon stimulation with the chemokine SDF-1, the remaining P-ERM proteins show a marked accumulation in the uropod, partially colocalizing with flotillin-2 (fluorescence pictures: Figure 1F). Of the stimulated T cells with accumulation of flotillin-2 in the uropod, 59 ± 9% (*n* = 3) also have P-ERM caps whereas 98 ± 1% of the T cells with P-ERM caps have flotillin-2 caps. These results are in agreement with our previous data in human T-lymphoblasts (Affentranger et al., 2011). The data suggest that in chemokine-stimulated cells only a small fraction of the ERM proteins is in the activated phosphorylated state and that activated P-ERM proteins preferentially accumulate in the uropod.

### T cell polarity is affected by the activation state of ezrin

**Figure 2:**
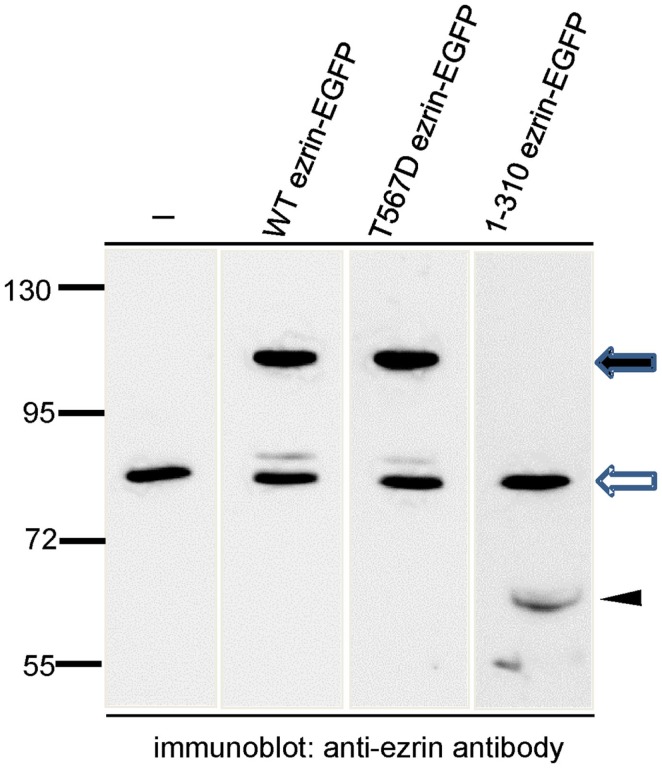
**Analysis of expression of wild type and mutant ezrin constructs in human T cells in relation to endogenous ezrin**. T cells were transiently transfected with the indicated constructs and EGFP-expressing T cells were sorted by FACS. The sorted cells were precipitated with TCA and subjected to immunoblotting, using an antibody reacting with the N-terminal domain of ezrin diluted 1:2000. The open arrow indicates endogenous ezrin (approximately 80 kDa); the closed arrow indicates the EGFP-tagged full-length proteins (approximately 110 kDa) and the arrowhead indicates the EGFP-tagged N-terminal ezrin fragment (approximately 60 kDa). Numbers at the left indicate molecular masses (kDa) of standard proteins. Quantitative evaluation of this blot indicates that WT ezrin-EGFP is overexpressed by 210% and T567D ezrin-EGFP by 335% as compared to endogenous ezrin. 1–310 ezrin-EGFP shows a substantially lower expression (approximately 10% of the endogenous ezrin).

**Figure 3:**
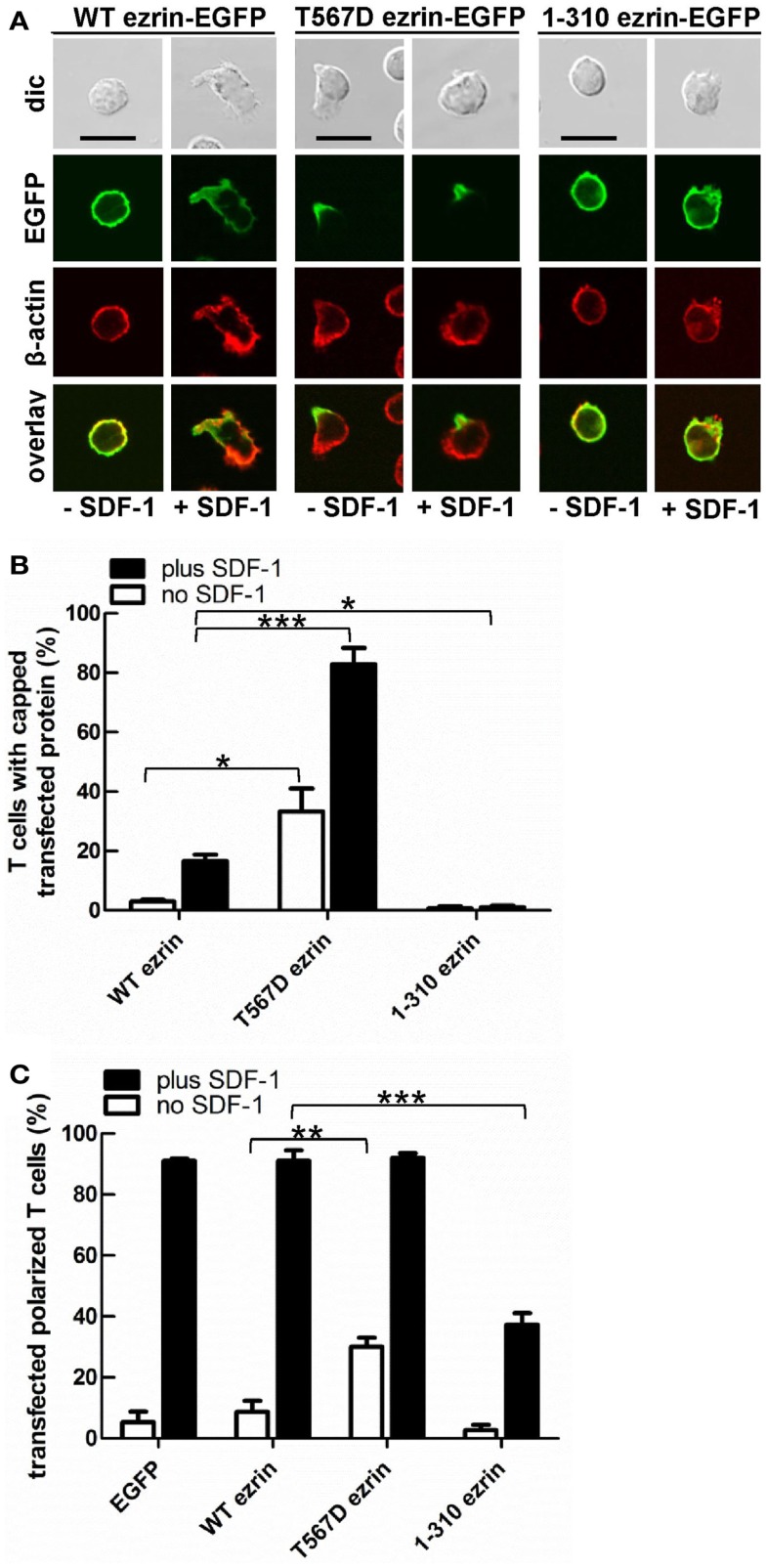
**Localization of tagged ezrin constructs in human T cells, and impact of expression of ezrin constructs on cell morphology**. **(A–C)** T-lymphocytes were transfected either with wild type (WT) ezrin-EGFP, T567D ezrin-EGFP, or 1–310 ezrin-EGFP. Four hours after transfection, cells were incubated in the absence or presence of SDF-1, as described in the legend of Figure 1D, followed by fixation with PFA and staining for β-cytoplasmic actin. Bar, 10 μm. **(A)** Fluorescence pictures obtained by confocal microscopy; **(B)** quantitative evaluation of the % of transfected T cells with capped EGFP-tagged wild type or mutant ezrin (mean ± SEM of three to five independent experiments); **(C)** quantitative evaluation of the % of transfected T cells with front-tail polarity (mean ± SEM of three to four experiments; exception: EGFP: *n* = 2). **(B,C)** *P < 0.05; **P < 0.01; ***P < 0.001 for statistically significant differences between data as indicated.

In order to test the role of ERM proteins in polarization and uropod scaffolding, we interfered with the functions of the endogenous ERM proteins by transiently transfecting freshly isolated human T cells with ezrin WT or mutants, all tagged at the C-terminus with EGFP. T567D ezrin mimics the phosphorylated form and is thus constitutively active. Conversely, the N-terminal 1–310 ezrin truncation mutant encodes only the FERM domain, and has widely been used to interfere with the function of all ERM proteins, presumably by uncoupling receptors from the cortical actin network (Martin et al., 1995; Faure et al., 2004). The amounts of expressed tagged proteins relative to the endogenous ezrin are documented in Figure 2. The majority (95 ± 3%) of cells transiently transfected with EGFP alone are spherical in the absence of the chemokine SDF-1. Addition of SDF-1 induces T cell polarization in 91 ± 1% of the EGFP-expressing cells (Figures 3A,C). WT ezrin-EGFP shows a uniform peripheral location in resting cells. In chemokine-stimulated cells, WT ezrin-EGFP shows a cortical but also cytosolic diffuse location (Figure 3A). In a small fraction (16 ± 2%) of the cells, WT ezrin-EGFP is capped in the uropod (Figure 3B). Quantitative evaluation of these experiments shows that transfection with WT ezrin-EGFP, as compared to EGFP, does not modify cell polarization in the absence or presence of SDF-1 (Figure 3C). Interestingly, however, transfection of T cells with the constitutively active T567D ezrin-EGFP induces cell polarization in 33 ± 8% of the transfected cells even in the absence of SDF-1. In these polarized T cells, T567D ezrin-EGFP localizes exclusively in the contracted uropod, opposite to β-actin-rich ruffles (Figures 3A,C). Transfection of T567D ezrin-EGFP does not further modify T cell polarization in SDF-1-stimulated cells. Conversely, the dominant-negative 1–310 ezrin mutant shows a random peripheral location and does not cap at all upon SDF-1-stimulation. Moreover, this construct markedly inhibits SDF-1-induced cell polarization by 57 ± 7% (*n* = 3) (Figure 3C). T cells expressing the 1–310 ezrin mutant and stimulated with SDF-1 still exhibit some β-actin-rich ruffles, with little colocalization of the ezrin construct and β-actin (Figure 3A). These findings strongly suggest that ERM protein function is needed for efficient chemokine-induced T cell polarization, and that in some cells, expression of active ERM proteins is sufficient to induce uropod formation.

### Ezrin activation state impacts the uropod localization of flotillins and PSGL-1

**Figure 4:**
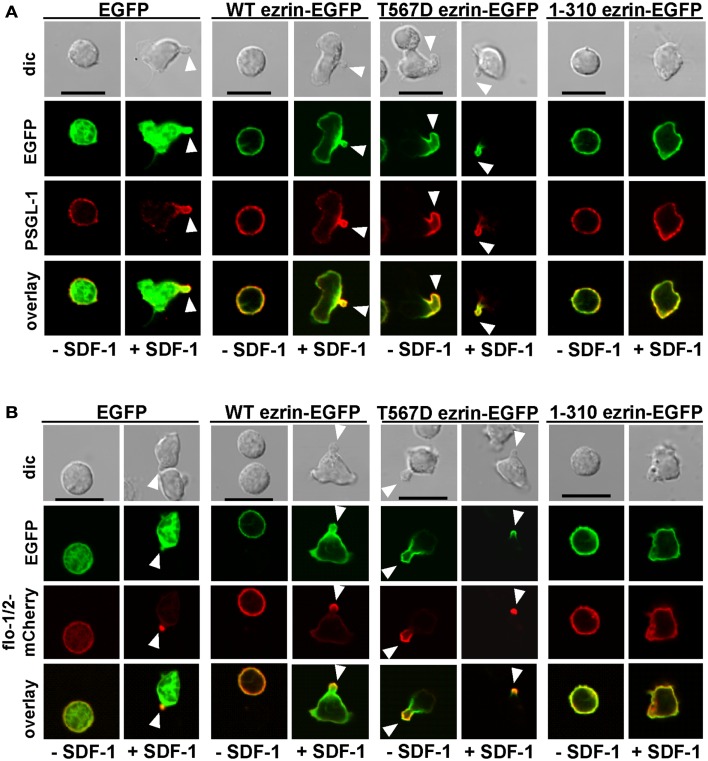
**Impact of expression of ezrin constructs on localization of PSGL-1 and flotillins in human T cells**. **(A)** T cells were transfected either with EGFP, wild type (WT) ezrin-EGFP, T567D ezrin-EGFP, or 1–310 ezrin-EGFP as indicated. Four hours after transfection, T cells were incubated in the absence or presence of SDF-1, as described in the legend of Figure 1D, followed by fixation with PFA, staining for PSGL-1 using a monoclonal antibody and confocal microscopy. Bar, 10 μm. **(B)** All T cells were cotransfected with flotillin-1-mCherry and flotillin-2-mCherry (singly expressed flotillins do not cap) and either with EGFP, wild type ezrin-EGFP, T567D ezrin-EGFP, or 1–310 ezrin-EGFP as indicated. Four hours after transfection, T cells were incubated in the absence or presence of SDF-1 as described in the legend of Figure 1, followed by fixation with PFA. Bar, 10 μm. White arrowheads indicate contracted uropods.

**Figure 5:**
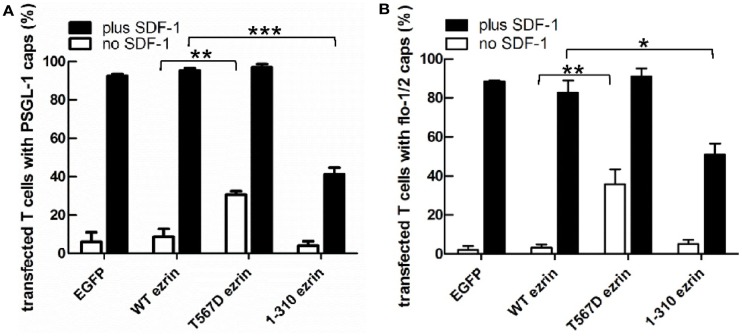
**Impact of expression of ezrin constructs on localization of PSGL-1 and flotillins in human T cells: quantitative evaluation**. **(A)** The fraction of transfected T cells with PSGL-1 caps was evaluated for the experiment shown in Figure 4A (mean ± SEM of three to four experiments, exception: EGFP: *n* = 2). **(B)** The fraction of transfected T cells with flotillin-mCherry caps was evaluated for the experiment shown in Figure 4B (mean ± SEM of four to five experiments, exception: EGFP: *n* = 2). Open bars: incubation for 30 min without SDF-1; closed bars: stimulation with SDF-1 for 15 min. *P < 0.05; **P < 0.01; ***P < 0.001 for statistically significant differences between data as indicated.

As previously shown, flotillins are required for efficient T cell uropod formation and capping of PSGL-1. Capping of flotillins and PSGL-1 correlates with cell polarization (Affentranger et al., 2011). Since transfection with T567D ezrin induces T cell front-tail polarity and formation of F-actin-rich ruffles, and 1–310 ezrin suppresses cell polarization, we were interested to know whether these mutants also impact uropod capping of flotillins and PSGL-1. As shown in Figures 4 and 5, the majority of T cells transiently transfected with EGFP alone are spherical and lack flotillin and PSGL-1 caps in the absence of SDF-1. Note that EGFP enters the nucleus, whereas the ezrin constructs are excluded from the nucleus. Addition of chemokine induces uropod capping of PSGL-1 and flotillins in 92 ± 1 and 94 ± 5% of the T cells respectively (Figure 4, quantified in Figure 5). Transfection with WT ezrin-EGFP, as compared to EGFP, does not modify localization of PSGL-1 and flotillins in the absence or presence of SDF-1. In contrast, transfection of cells with the constitutively active T567D ezrin-EGFP significantly induces flotillin and PSGL-1 capping in 32 ± 2% of the transfected T cells even in the absence of SDF-1. Capping of flotillins and PSGL-1 occurs only in T cells with capped T567D ezrin. In some cells expressing T567D ezrin, the uropod is enlarged, comparable to the findings of Lee et al. (2004). Expression of T567D ezrin-EGFP does not further modify protein capping in SDF-1-stimulated T cells as compared to T cells expressing WT ezrin. T567D ezrin-EGFP shows a marked colocalization with PSGL-1 and flotillins in the uropods. Conversely, dominant-negative 1–310 ezrin-EGFP partially prevents capping of flotillin and PSGL-1. Specifically, capping of PSGL-1 is inhibited by 54 ± 5% (*n* = 3), as compared to T cells transfected with WT ezrin. The effect on flotillin capping is smaller but also statistically significant (inhibition: 35 ± 9%, *n* = 4). Thus, expression of constitutively active ezrin induces front-tail polarity and capping of flotillins and PSGL-1 to a similar extent, whereas the dominant-negative ezrin mutant has a comparable inhibitory effect on uropod formation and capping of uropod proteins.

### siRNA-mediated suppression of flotillin-1 and -2 in murine T-lymphoblasts impairs moesin capping and uropod formation

**Figure 6:**
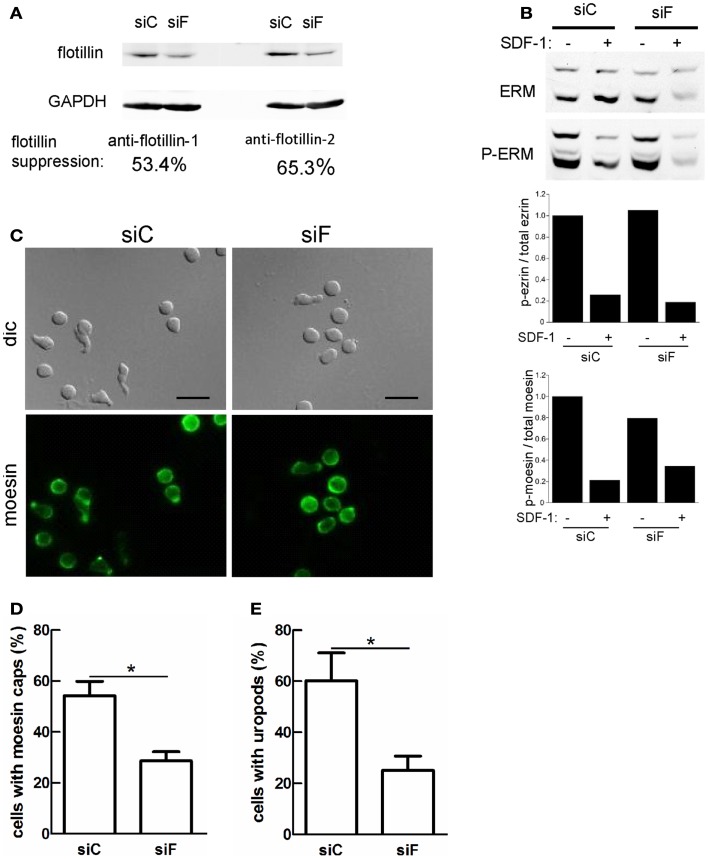
**Reduction of flotillin-1 and -2 expression in murine T-lymphoblasts impairs moesin capping and uropod formation**. Murine T-lymphoblasts were treated with control siRNA (*siC*) or siRNA directed against murine flotillin-2 (siF). **(A)** Downregulation of both flotillin-1 and -2 was assessed by immunoblotting. **(B)** Murine T-lymphoblasts prepared as in **(A)** were untreated or treated with 10 nM SDF-1α for 15 min, and lysates were immunoblotted for total or P-ERM proteins. Relative phosphorylation of ezrin and moesin was obtained by densitometry (ratio of values obtained for phosphorylated moesin or ezrin, divided by values obtained for total moesin or ezrin, and normalized to the control sample). Data are from one experiment, representative of three. Note that the low signal in the siF +SDF-1 lane is due to underloading and is not reproducibly observed. **(C–E)** T cells prepared as in **(A)** were allowed to adhere to fibronectin-coated glass, fixed and stained for moesin **(C)** and the % of cells with moesin caps **(D)** and uropods **(E)** was evaluated. Bar, 10 μm. **(D,E)** Mean ± SEM of four experiments; *P < 0.05.

We showed previously that transfection of human T-lymphoblasts with a dominant-negative mutant of flotillin-2 impairs cell polarization and capping of endogenous flotillin-1 and PSGL-1 (Affentranger et al., 2011). We therefore asked whether interference with flotillin expression also modifies capping of activated ERM proteins. To this end, we performed siRNA-mediated suppression of flotillin-2 expression. These experiments were done in murine T cells, since flotillin suppression in these cells was more efficient than in human T cells where we could achieve maximally approximately 30% downregulation. Using flotillin-2 specific siRNA in murine T-lymphoblasts, expression of both flotillin-1 and -2 could be suppressed by about 53 and 65%, respectively (Figure 6A). This agrees with previous findings in other cell types, that the stability of flotillin-1 depends on that of flotillin-2 (Solis et al., 2007). ERM protein levels are unaffected, demonstrating specificity of suppression (Figure 6B). In murine T cells, about 50% of the ERM proteins are phosphorylated (Shaffer et al., 2009) and flotillin suppression has no effect on basal levels of ERM phosphorylation or on patterns of chemokine-stimulated ERM dephosphorylation (Figure 6B and data not shown). Moesin shows appreciable capping in T-lymphoblasts adhering to fibronectin-coated glass (Figure 6C). Moesin capping is significantly reduced by 46 ± 9% (*n* = 4) in cells with reduced flotillin expression. Similarly, uropod formation is reduced by 56 ± 8% (*n* = 4) in cells with downregulated flotillins (Figures 6D,E), indicating that flotillins and ERM proteins cooperate in uropod scaffolding and formation.

### Uropod formation induced by T567D ezrin is perturbed by disruption of the acto-myosin cytoskeleton in human T cells

**Figure 7:**
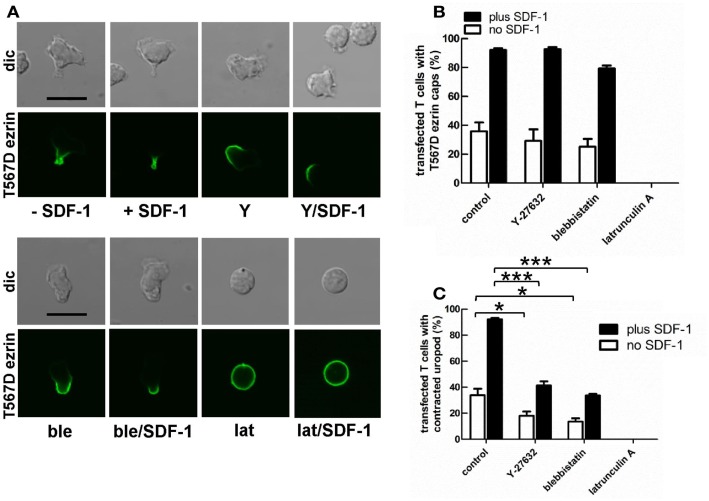
**Impact of cytoskeletal perturbation on capping of T567D ezrin-EGFP and on human T cell front-tail polarity induced by T567D ezrin-EGFP**. **(A–C)** T cells were transfected with T567D ezrin-EGFP. Four hours after transfection, cells were preincubated in the absence or presence of 20 μM Y-27632 (Y) (to inhibit Rho-kinase) or 50 μM blebbistatin (ble) (to inhibit myosin II) or 1 μM latrunculin A (lat) (to disrupt F-actin), followed by a further incubation in the absence or presence of SDF-1 (40 ng/ml) for 15 min. T cells were then fixed with PFA, followed by confocal microscopy **(A)**. The % of transfected cells with T567D ezrin-EGFP caps **(B)** and the % of transfected cells with contracted uropods **(C)** was evaluated. Bar, 10 μm **(B,C)**: mean ± SEM of three to seven independent experiments). *P < 0.05; ***P < 0.001 for statistically significant differences between data as indicated.

**Figure 8:**
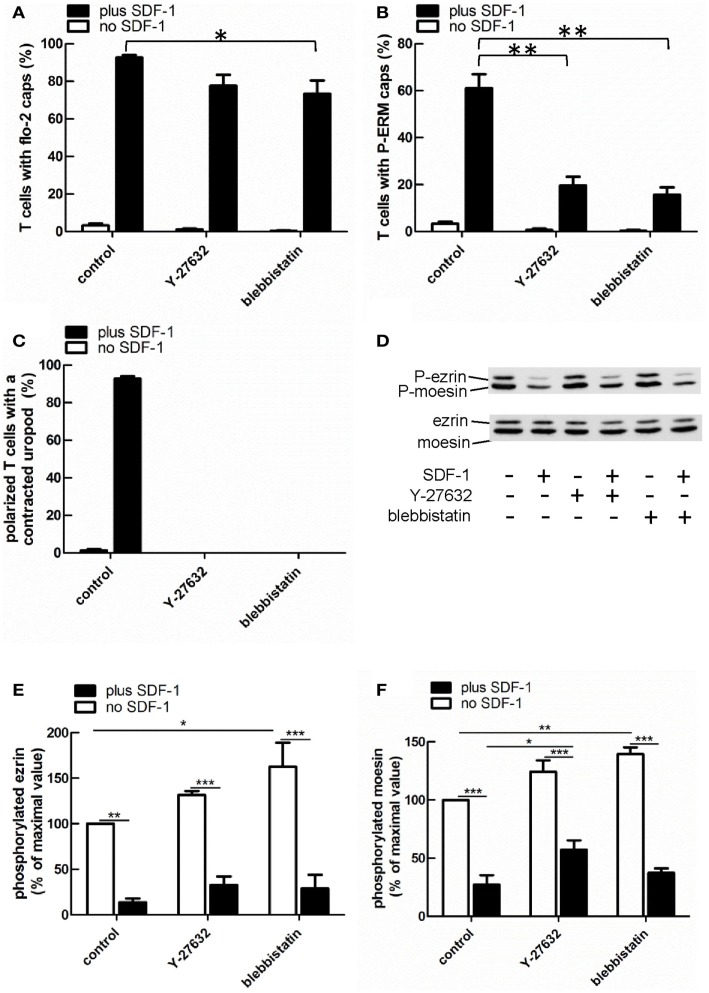
**Impact of cytoskeletal perturbation on capping of endogenous flotillin-2 and P-ERM and on human T cell front-tail polarity induced by SDF-1**. **(A–C)** T cells were preincubated for 30 min in the absence or presence of 20 μM Y-27632 or 50 μM blebbistatin or 1 μM latrunculin A, followed by a further incubation in the absence or presence of SDF-1 (40 ng/ml) for 15 min. T cells were then fixed with TCA and stained for flotillin-2 and P-ERM. The % of T cells with flotillin-2 caps **(A)** and P-ERM caps **(B)** and the % of cells with front-tail polarity featuring contracted uropods **(C)** was evaluated (mean ± SEM of three independent experiments). *P < 0.05, **P < 0.01, for statistically significant differences between data as indicated. **(D–F)** T cells were treated with Y-27632 or blebbistatin and SDF-1 as described for **(A–C)**, followed by precipitation with TCA and immunoblotting using an antibody recognizing P-ERM, and as loading control, an antibody recognizing all ERM proteins independent of phosphorylation. **(D)** shows a representative immunoblot. **(E,F)** Quantitative evaluation of the experiment shown in **(D)** obtained by densitometry. (Ratio of values obtained for phosphorylated moesin or ezrin, divided by values obtained for total moesin or ezrin). Mean ± SEM of three to four independent experiments. *P < 0.05; **P < 0.01; ***P < 0.001 for statistically significant differences between data as indicated.

Myosin contractility, activated by Rho-kinase activity, is essential for uropod contraction in chemokine-stimulated T-lymphoblasts, but is not required for capping of flotillins and PSGL-1 (Affentranger et al., 2011). To test the relationship between polarization induced by constitutively active T567D ezrin and the acto-myosin cytoskeleton, we used Y-27632, blebbistatin, and latrunculin A as inhibitors of Rho-kinase, myosin II, and F-actin, respectively. Treatment of human T cells with Y-27632 or blebbistatin does not significantly affect cap formation of T567D ezrin-EGFP in the absence or presence of SDF-1 (Figures 7A,B). However formation of a contracted uropod in cells transfected with T567D ezrin-EGFP in the absence or presence of SDF-1 is inhibited significantly by approximately 50% by these inhibitors (Figures 7A,C), indicating a role of myosin II and Rho-kinase. We also investigated the impact of Y-27632 and blebbistatin on capping of endogenous P-ERM and flotillin-2. As shown in Figure 8A, endogenous flotillin caps in SDF-1-stimulated freshly isolated T cells are relatively resistant to these inhibitors (16 ± 6% reduction of flotillin-2 caps by Y-27632; 21 ± 7% reduction by blebbistatin). In contrast, SDF-1-induced capping of endogenous P-ERM proteins is markedly reduced (Figure 8B; 67 ± 8% inhibition by Y-27632; 73 ± 7% inhibition by blebbistatin). Moreover, SDF-1-induced formation of contracted uropods is completely abolished by both inhibitors (Figure 8C). The inhibitory effects of Y-27632 and blebbistatin on P-ERM capping could be explained by a reduction in ERM phosphorylation in cells treated with these drugs. However, as shown in Figures 8D–F, preincubation of T cells with either Y-27632 or blebbistatin does not prevent P-ERM dephosphorylation. Indeed, we observed significant increases in phosphorylation of both ezrin and moesin both before and after chemokine addition. Thus, as reported previously (Belkina et al., 2009), Rho-kinase does not appear to play an important role in phosphorylating T cell ERM proteins. Rather, both active Rho-kinase and myosin II appear to negatively regulate ERM phosphorylation. On the basis of these results, we conclude that Rho-kinase and myosin activity are required for uropod formation independently of ERM phosphorylation.

Prevention of actin polymerization by treatment of T cells with latrunculin A completely abolishes both T567D ezrin cap formation and uropod formation in the absence and presence of SDF-1, indicating an important role of the cortical F-actin in capping of overexpressed constitutively active ezrin and in induction of polarization (Figure 7). In order to obtain more information on the role of actin in capping and immobilization of ezrin, we analyzed the lateral mobility of T567D ezrin-EGFP, as compared to WT ezrin-EGFP, in T cells using FRAP. Note that these FRAP experiments were carried out at 20°C, as rapid shape changes and migration occurring at 37°C precluded accurate bleaching, acquisition and evaluation of FRAP data. The T cells were cotransfected with flotillin-1 and -2 tagged with mCherry as uropod markers (singly expressed flotillins do not cap, see Baumann et al., 2012) and bleaching of EGFP was carried out in the region of flotillin caps, if present. As shown in Table 1, 75% of T567D ezrin-EGFP is immobilized in cells not exposed to SDF-1, as compared to only 26% of WT ezrin. Mobility of T567D ezrin-EGFP is significantly (*P* < 0.01) restored by pretreatment with latrunculin A to values obtained for WT ezrin, indicating immobilization due to F-actin interaction. Similar data were obtained for cells treated with SDF-1. In contrast, the majority of the WT ezrin-EGFP molecules are mobile, irrespective of the presence or absence of latrunculin A or SDF-1 (Table 1). This finding correlates with the observation that WT tagged ezrin does not cap significantly in the uropod in SDF-1-stimulated cells (Figure 3). Thus only a small fraction of total ezrin appears to be phosphorylated/activated and capable of interacting with F-actin.

**Table 1:** **Lateral mobility of wild type ezrin and T567D ezrin-EGFP assessed with fluorescence recovery after photobleaching (FRAP) in human T cells^a^**.

Ezrin construct	Pretreatment	*M*_F_ − SDF-1 (*n*)	*M*_F_ + SDF-1 (*n*)
WT ezrin-EGFP	No pretreatment	0.74 ± 0.06 (10)	0.64 ± 0.11 (6)
WT ezrin-EGFP	Latrunculin A (1 μM, 30 min)	0.81 ± 0.06 (10)	0.79 ± 0.07 (10)
T567D ezrin-EGFP	No pretreatment	0.25 ± 0.05 (11)	0.18 ± 0.02 (10)
T567D ezrin-EGFP	Latrunculin A (1 μM, 30 min)	0.72 ± 0.06 (10)	0.81 ± 0.03 (9)

*^a^Human T cells were cotransfected with wild type (WT) ezrin or T567D ezrin-EGFP and with flotillin-1 and -2 tagged with mCherry. FRAP analysis involving local bleaching of EGFP in the area of the flotillin caps was carried out as described in the Section “Materials and Methods” with T cells preincubated in the absence or presence of SDF-1 (15 min, 40 ng/ml) and without or with pretreatment with latrunculin A as indicated. The mobile fraction (*M*_F_) was calculated for each individual cell and then averaged from a total of *n* = 9–11 cells observed in three independent experiments (mean ± SEM)*.

### Expression of T567D ezrin promotes T cell migration in the absence of added chemokine, whereas 1–310 ezrin attenuates this process

**Figure 9:**
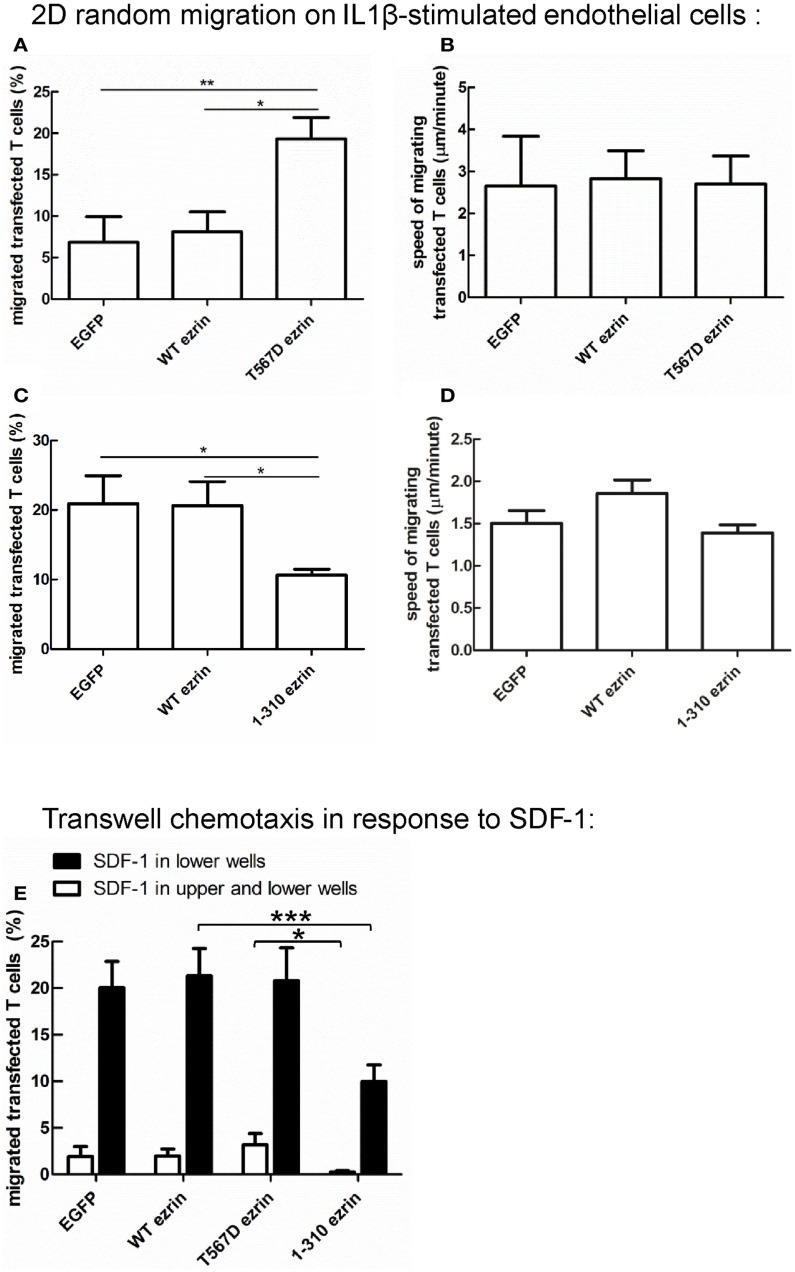
**Impact of expression of ezrin constructs on random human T cell migration and chemotaxis**. **(A,B)** T cells were transfected with EGFP or wild type ezrin or T567D ezrin or 1–310 ezrin as indicated and placed 4 h later on a layer of IL1β-stimulated human endothelial cells in the absence of added chemokine. Random migration of the transfected T cells was assessed for 10 min by time-lapse videomicroscopy at 37°C. The % of migrating transfected T cells **(A,C)** and the mean speed of the migrating transfected T cells **(B,D)** was evaluated. Mean ± SEM of three experiments. *P < 0.05, **P < 0.01 for statistically significant differences between data as indicated (total number of T cells analyzed in three experiments: 247/656 cells transfected with EGFP; 376/574 cells transfected with wild type ezrin; 437 cells transfected with T567D ezrin; 679 cells transfected with 1–310 ezrin). **(E)** T cells were transfected with the indicated constructs and placed 4 h later into filter inserts. The lower and upper wells (open bars) or only the lower wells (closed bars) contained 200 ng/ml SDF-1. The number of transfected migrated T cells in the lower wells was determined after 1 h and calculated as % of total transfected T cells placed initially into the upper wells. In the absence of SDF-1 in the lower wells migration was zero for all samples and experiments. Mean ± SEM of eight experiments for SDF-1 only in the lower wells and of three experiments for SDF-1 in the upper and lower wells. *P < 0.05, ***P < 0.001 for statistically significant differences between data as indicated.

Cell polarization is a prerequisite for migration. Since expression of T567D ezrin enhances cell polarization and that of 1–310 ezrin attenuates polarization, we assessed the impact of these constructs on migration, in comparison to WT ezrin. For this purpose, we measured random spontaneous 2D T cell migration in the absence of added chemokine on IL1β-stimulated human endothelial cell layers, which express adhesion molecules such as ICAM-1. As shown in Figure 9A, only 7 ± 3% of T cells expressing EGFP and 8 ± 2% of T cells expressing WT ezrin migrate in this assay, whereas 19 ± 3% of the T cells expressing T567D ezrin are migratory. Analysis of the fractions of migrating cells revealed a significant 2.7 ± 0.6-fold increase (*n* = 3, *P* < 0.05) when comparing T cells expressing WT ezrin and T567D ezrin, and a 4.3 ± 1.6-fold increase (*n* = 3, *P* < 0.01) when comparing T cells expressing EGFP and T567D ezrin. The mean speed of the migrating T cells is not significantly different for cells transfected with any of the ezrin constructs (Figure 9B). T cells expressing T567D ezrin and spontaneously migrating on IL1β-stimulated endothelial cell layers in the absence of chemokine are shown in Movie S1 in Supplementary Material. Occasionally we observed migrating cells that simultaneously changed their polarity, as well as the position of the T567D ezrin cap in the rear and the direction of migration during the movie. One such cell is shown in this movie (cell on the right).

In a separate series of experiments, we assessed the impact of 1–310 ezrin expression in comparison to T cells transfected with EGFP or WT ezrin. As shown in Figure 9C, transfection of T cells with 1–310 ezrin results in a 45 ± 11% (*n* = 3, *P* < 0.05) reduction in the fraction of migrating cells as compared to cells transfected with WT ezrin, and a 44 ± 16% (*n* = 3, *P* < 0.05) reduction as compared to T cells transfected with EGFP. As with cells expressing the constitutively active ezrin, migrating cells expressing the dominant-negative 1–310 mutant do not show significant changes in speed (Figure 9D). Note that in the second series of experiments shown in Figures 9C,D, the fraction of spontaneously migrating cells transfected with EGFP or WT ezrin was somewhat increased as compared to the first series shown in Figures 9A,B due to biological variations in the experimental system.

### Expression of 1–310 ezrin attenuates T cell chemotaxis

In addition to analyzing random cell migration, we also investigated SDF-1-induced chemotaxis. Transfection of human T cells with WT or T567D ezrin does not significantly modify SDF-1-induced migration through 3 μm transwell filters as compared to transfection with EGFP alone (Figure 9E). In contrast, transfection with 1–310 ezrin reduces chemotaxis significantly (inhibition: 53 ± 5%, *n* = 8, as compared to cells expressing WT ezrin) (Figure 9E), in agreement with the findings for 2D migration (Figures 9C,D). These data correlate with the negative effect of this construct on cell polarity (Figure 3). The reduction in chemotaxis as a result of 1–310 ezrin expression is similar in magnitude to the reduction in polarized cells and cells with capped flotillins (Figures 3–5). Little migration is observed when SDF-1 is present both in the upper and the lower wells, indicating predominant chemotactic (and not chemokinetic) migration (Figure 9E).

## Discussion

We provide here novel data showing that expression of activated ezrin in primary human T cells is sufficient to induce polarization responses involving formation of a contracted uropod enriched in membrane-microdomain-associated flotillins and the adhesion receptor PSGL-1, as well as a leading edge bearing β-actin-rich ruffles. These T cells are capable of migration in the absence of added chemokine and chemotax normally in a gradient of SDF-1. T567D ezrin does not significantly modify polarization or chemotaxis of SDF-1-stimulated cells. Conversely, dominant-negative 1–310 ezrin had no impact on the morphology of resting cells, but significantly reduced polarization, capping of flotillins and PSGL-1 and chemotaxis of SDF-1-stimulated cells. These data strongly suggest an important positive role of ERM proteins in self-organizing T cell polarity, particularly with respect to uropod formation and uropod recruitment of adhesion receptors and membrane-microdomain scaffolding proteins such as flotillins. We also show that downregulation of flotillins in murine T cells impairs moesin capping and uropod formation without perturbing ERM protein phosphorylation. Together, these findings suggest that ERM proteins and flotillins mutually enhance uropod capping and cooperate in uropod formation. Our findings are in agreement with data obtained for a constitutively polarized murine lymphoma cell line, where transfection with T567D ezrin increased uropod size, whereas 1–310 ezrin reduced the fraction of cells with uropod (Lee et al., 2004). Interestingly, in that study T567D ezrin induced capping of CD44, but not uropod formation in a naturally unpolarized myeloma cell line, indicating that in these cells the machinery for uropod formation is lacking, in contrast to the T cells used in our study.

We recently investigated chemotaxis, adhesion, and homing of murine T-lymphoblasts lacking ezrin and with markedly reduced moesin expression (Chen et al., 2013). In agreement with this work on naive human T cells, *in vitro* chemotaxis to CCL19 of these T-lymphoblasts deficient in ERM proteins through 3 μm pores was reduced by 20–50%. However no difference to WT cells could be detected concerning chemotaxis through 5 μm pores or migration in collagen gels. Defects in front-tail polarity could only be detected in ERM-deficient T-lymphoblasts plated on β1-integrin ligands, but not on β2-integrin ligands. Possibly the small amounts of residual moesin in these cells may explain the moderate phenotype of these T-lymphoblasts. Alternatively, naive T cells may rely more on ERM proteins than T-lymphoblasts.

Liu et al. (2012) investigated the migration of murine T-lymphoblasts cultured from transgenic mice expressing low levels of phosphomimetic T567E ezrin. There, in contrast to our data, expression of T567E ezrin reduced cell polarization and protrusion formation. This discrepancy may be due to differences in the extent of overexpression and/or to the use of murine expanded T-lymphoblasts (Liu et al., 2012) versus freshly isolated human T cells (this work). Liu et al. (2012) observed an increase in membrane tension in T cells expressing T567E ezrin (about 50% of endogenous ezrin) and proposed that this was responsible for reducing protrusion formation. In contrast, we found that transfection of human T cells with T567D ezrin (about 335% of endogenous ezrin) actually enhanced protrusion and polarization. In our hands, polarity was observed only in cells with capped T567D ezrin; not in cells with randomly located T567D ezrin. In the cells with capped T56D ezrin, rigidity of the cell cortex might be enhanced locally, so that the formation of protrusions is suppressed in one region of the cell. This still would allow or possibly even promote localized protrusion formation on the opposite side of the cap where T567D ezrin is lacking (Figure 3). Possibly, substantial capping of T567D ezrin only occurs at higher expression levels. Further, Liu et al. (2012) report that *in vitro* chemotaxis and migration of murine T-lymphoblasts expressing T567E ezrin on ICAM-1 coated surfaces was inhibited by about 20% as compared to cells transfected with WT ezrin. In our study we did not observe a significant difference in *in vitro* chemotaxis between human T cells expressing T567D ezrin as compared to cells transfected with WT ezrin (Figure 9).

We show that although formation of a contracted uropod requires an intact cytoskeleton and myosin II activity, spontaneous capping of T567D ezrin requires F-actin but not myosin II activity or Rho-kinase activity (Figure 7). The requirement for F-actin may be explained by spontaneous actin flow, which may promote accumulation of T567D ezrin at one side of the cell. Retrograde F-actin flow has been shown to occur in T cells independently of myosin II activity (Babich et al., 2012). Indeed, T567D ezrin appears to be immobilized by interaction with F-actin, as shown by our FRAP experiments, in contrast to WT ezrin which is much more mobile (Table 1). Uropod formation induced by T567D ezrin in the absence of chemokine is markedly reduced (but not abolished) by inhibition of Rho-kinase or myosin II activity (Figure 7). Similarly, inhibition of Rho-kinase or myosin II impairs uropod formation in SDF-1-stimulated T cell without reducing phosphorylation of endogenous ERM proteins (Figure 8). This indicates that Rho-kinase and myosin II activity are required for uropod contraction independently of the state of ERM phosphorylation. Constitutively active T567D ezrin could locally enhance Rho activity and thus myosin II activity and uropod contraction, in line with previous findings (Ivetic and Ridley, 2004; Lee et al., 2004; Luo et al., 2012). In addition, localized Rho activation in the T567D ezrin caps may locally inactivate Rac, resulting in relative Rac activation in other parts of the cell that then would promote formation of F-actin-rich protrusions in the front. A similar process has been proposed to occur in zebrafish neutrophils treated with microtubule depolymerizing agents that induce Rho activation and promote polarization and migration (Yoo et al., 2012).

Based on the currently available data, we postulate that the following sequence of events occurs during chemokine-induced T cell polarization: in resting T cells, uniformly membrane-associated P-ERM prevents the formation of protrusions at the plasma membrane by stabilizing a cortical actin network. Activation induces dephosphorylation of ERM proteins and dissociation from the cortical actin, allowing formation of protrusions in all directions. Later, the remaining activated ERM proteins accumulate in the uropod driven by actin flow, where they locally reinforce cortical F-actin, prevent protrusions, activate Rho/contractility, and act as scaffolding proteins, together with flotillins. In this context it is interesting that data obtained for neutrophils derived from mice lacking flotillin-1 indicate a role for flotillin microdomains in enhancing myosin II activity (Ludwig et al., 2010). Other proteins may also contribute to T cell uropod scaffolding, for example polarity proteins such as Scribble, Par3, and Crumbs complexes that regulate epithelial polarity. Interestingly proteins from these complexes are also expressed in T cells, and downregulation of expression of Scribble in the MD45 T cell line results in reduction of uropod formation, ERM uropod location and migration (Ludford-Menting et al., 2005).

Concerning the relationship between flotillins and P-ERM, our data suggest that these proteins mutually reinforce their uropod location by an as yet unknown mechanism. Based on our data, enhancement of P-ERM uropod enrichment by flotillins does not involve a role of flotillins in regulating ERM phosphorylation. We tested whether flotillins and ERM proteins co-immunoprecipitate using lysates of human T cells but could not find any positive evidence for this (Baumann and Niggli, unpublished observations). A possible link between flotillins and ERM proteins could be the transmembrane receptor PSGL-1, a well established interaction partner of ERM proteins (Ivetic and Ridley, 2004). We obtained evidence for flotillin-PSGL-1 interactions in human neutrophils using co-immunoprecipitation (Rossy et al., 2009). Moreover our recent findings using the proximity ligation assay (PLA) also suggest such an interaction to occur in freshly isolated human T cells. The *in situ* PLA is based on modified secondary antibodies reacting with murine or rabbit IgG. These secondary antibodies are conjugated with different oligonucleotides. Annealing of the probes occurs when the target proteins are in close proximity (less than 30–40 nm distance), which then initiates the amplification. The amplicons can be detected by fluorescence microscopy (Söderberg et al., 2006). Using polyclonal rabbit antibodies directed to flotillin-2 and murine monoclonal antibodies directed to PSGL-1 we detected PLA amplicons randomly distributed along the plasma membrane in 81–83% of freshly isolated human T cells incubated in the absence of chemokine. In 82–93% T cells stimulated for 15 min with SDF-1 the amplicons were concentrated in the uropod, at the plasma membrane, whereas in controls only using the anti-flotillin-2 antibody, fluorescent dots could be detected in less than 5% of the T cells (Baumann et al., unpublished observations). Thus flotillins could recruit PSGL-1 which in turn would enhance capping of P-ERM, and vice versa. Another link could be a lipid intermediate. ERM proteins interact with PIP-2-rich membranes (Niggli and Rossy, 2008), and a significant pool of ERM proteins has been detected in lipid raft fractions of T cells (Tomas et al., 2002) where flotillins also reside (Rajendran et al., 2009). Cholesterol-rich microdomains have been shown to be enriched in the uropod of human T-lymphoblasts, and disruption of these microdomains by methyl-β-cyclodextrin disrupt uropod formation and impair chemotaxis (Millán et al., 2002). The possible role of flotillins in modulating recruitment of ERM and other uropod proteins such as PSGL-1 to uropod rafts requires further studies. It has to be noted that inhibition of Rho-kinase or myosin II in SDF-1-stimulated T cells markedly reduces capping of endogenous P-ERM by 67–73%, without inhibiting ERM phosphorylation, but only reduces capping of endogenous flotillin-2 by 16–21%. Thus, although transfection with T567D ezrin is sufficient to induce capping of flotillins, endogenous flotillins can cap without concomitant P-ERM capping. In keeping with this, 93% of the chemokine-stimulated T cells have flotillin-2 caps in the uropod, but only 61% exhibit P-ERM caps (Figure 8). Thus, it appears that activated ERM proteins are not absolutely required for flotillin uropod capping and that flotillin capping does not obligatorily induce P-ERM capping.

The N-terminal cargo-binding domain of 1–310 ezrin inhibits ERM protein function in many systems, and here we find that it inhibits T cell polarity responses. This truncated protein may work in several ways. Faure et al. (2004) reported that transfection of T cells with 1–310 ezrin causes dephosphorylation of endogenous ERM proteins and uncoupling of actin from the plasma membrane. In addition, this construct has the potential to disrupt other processes not directly regulated by ERM proteins. For example, 1–310 ezrin could interact with PIP-2 and prevent its interaction with other targets. Note that this construct has significant negative effects on T cell functions despite its relatively low level of expression (Figure 2). Possibly this can be explained by only a small fraction of the total ERM protein pool being in the active form in human T cells (Figure 1), whose function can be inhibited by low levels of 1–310 ezrin.

In conclusion, we show that expression of constitutively active T567D ezrin in freshly isolated human T cells is sufficient to induce cell polarization, that is, formation of a contracted uropod enriched in flotillins and PSGL-1, and a front with F-actin-rich ruffles. These polarized cells show increased random migration in the absence of added chemokine. Uropod formation, but not ruffling, induced by T567D ezrin is partly dependent on myosin II/Rho-kinase activity. Conversely, transfection with dominant-negative 1–310 ezrin attenuates chemokine-induced polarization, capping of uropod proteins, spontaneous 2D migration, and chemotaxis. Our data indicate an important positive role of ERM proteins in polarization and migration of T cells and suggest that flotillins and ERM proteins cooperate in uropod formation. Open questions concern the exact roles of ERM proteins in uropod scaffolding and signaling and the mechanisms of cooperation between flotillins and ERM proteins.

## Conflict of Interest Statement

The authors declare that the research was conducted in the absence of any commercial or financial relationships that could be construed as a potential conflict of interest.

## Supplementary Material

The Supplementary Material for this article can be found online at http://www.frontiersin.org/T_Cell_Biology/10.3389/fimmu.2013.00084/abstract

Supplementary Movie S1 **Random migration of human T cells expressing constitutively active T567D ezrin-EGFP**. T cells were transfected with T567D ezrin-EGFP and placed 4 h later on an Il-1-beta-stimulated human endothelial cell layer in the absence of added chemokine. Random migration of the transfected T cells was assessed for 10 min by time-lapse videomicroscopy at 37°C (four images per min). The movie was created with eight frames per second (1 s of movie = 2 min real time).
